# Microbial β C-S Lyases: Enzymes with Multifaceted Roles in Flavor Generation

**DOI:** 10.3390/ijms25126412

**Published:** 2024-06-11

**Authors:** Mathieu Schwartz, Nicolas Poirier, Jade Moreno, Alena Proskura, Mélanie Lelièvre, Jean-Marie Heydel, Fabrice Neiers

**Affiliations:** 1 Center for Taste and Feeding Behavior, CNRS, INRAE, Institut Agro, University of Burgundy, F-21000 Dijon, Francefabrice.neiers@u-bourgogne.fr (F.N.); 2 International Research Center “Biotechnologies of the Third Millennium”, Faculty of Biotechnologies (BioTech), ITMO University, 191002 Saint-Petersburg, Russia

**Keywords:** β C-S lyase, flavor, aromatic thiol, aroma, cysteine S-conjugates, oral microbiota

## Abstract

β C-S lyases (β-CSLs; EC 4.4.1.8) are enzymes catalyzing the dissociation of β carbon–sulfur bonds of cysteine S-conjugates to produce odorant metabolites with a free thiol group. These enzymes are increasingly studied for their role in flavor generation in a variety of food products, whether these processes occur directly in plants, by microbial β-CSLs during fermentation, or in the mouth under the action of the oral microbiota. Microbial β-CSLs react with sulfur aroma precursors present in beverages, vegetables, fruits, or aromatic herbs like hop but also potentially with some precursors formed through Maillard reactions in cooked foods such as meat or coffee. β-CSLs from microorganisms like yeasts and lactic acid bacteria have been studied for their role in the release of polyfunctional thiols in wine and beer during fermentation. In addition, β-CSLs from microorganisms of the human oral cavity were shown to metabolize similar precursors and to produce aroma in the mouth with an impact on retro-olfaction. This review summarizes the current knowledge on β-CSLs involved in flavor generation with a focus on enzymes from microbial species present either in the fermentative processes or in the oral cavity. This paper highlights the importance of this enzyme family in the food continuum, from production to consumption, and offers new perspectives concerning the utilization of β-CSLs as a flavor enhancer.

## 1. Introduction

The sensory experience encountered during food consumption is considered the main factor in determining the acceptability and consumption of food. Among the various aspects of this experience, flavor perception is recognized as a critical component. A complex interplay of information gathered from the chemical senses, including olfaction, gustation, and somatosensory inputs from the nasal and oral regions, is encompassed by it. The integration of sensory information coming from the chemical composition of the food enables the assessment of its quality. However, it should be noted that the input of these chemical signals to taste and olfactory receptors can be influenced by the environment surrounding these receptors [1,2,3]. Investigations have evidenced the role played by enzymatic processes in modifying flavor compounds, subsequently impacting how these flavors are perceived [4,5,6,7,8]. Recently, it was shown that the metabolic activity of odorant compounds shapes the brain’s representations of the olfactory world [9]. As a result, the enjoyment of food and proper nutrition can be hindered by age-related salivary disorders, or health status such as obesity [10,11]. In addition to human oronasal metabolism, recent scientific inquiries have evidenced the intriguing influence exerted by the oral microbiota on the perception of taste and food preferences [12,13,14,15]. Importantly, specific microorganisms residing in the oral microbiota possess the capability to generate aromatic compounds within the mouth, actively participating in the food flavor [16,17,18]. These findings suggest that variations in the composition of these oral microbes could potentially contribute to differences in flavor perception. While these concepts might be perceived as groundbreaking in the field of chemoperception, it is important to note that some of the molecular mechanisms at play have been well-established in the field of food microbiology.

Food product fermentation has a profound impact on the sensory properties of the final products. Microorganisms have been harnessed by humans for millennia, initially through brewing and baking, and later in the production of bread, cheese, beer, wine, and yogurt. Fermentation is a biological process that not only transforms raw ingredients into an array of products but also adds depth and complexity to the global food flavor [19,20]. Microorganisms like bacteria and yeasts participate in fermentation, converting sugars and other compounds into a spectrum of flavor compounds. Fermentation intensifies flavors by adding new and complex profiles, which are valuable for their uniqueness and complexity. For example, microbial succession during the fermentation of sufu (fermented tofu) was shown to be important for the generation of a diversity of umami peptides [21]. Microbial metabolism can generate new flavor compounds from aromatic precursors that are non-odorant before being metabolized as well as reduce the bitterness of other compounds consequently acting on the flavor in a general manner [22,23]. Advances in food metabolomics, i.e., the large-scale study of metabolites within a food product, have evidenced the diverse array of metabolites generated by microorganisms during fermentation and proved to be valuable for food quality analysis [24]. It was shown that similarities exist in the enzyme families responsible for flavor generation both in the fermentation processes and in the mouth [12]. Microbial enzymes responsible for the generation of these new flavor compounds mainly include glycosidases and carbon–sulfur lyases. Glycosidases have been recently reviewed by Muradova and coworkers, showing the importance of this enzyme family in the generation of aromatic aglycone from non-aromatic glycoconjugates present in a variety of food products including plant-based milks, fruit juices, wines, and teas [25]. Additionally, glycosidase activities were measured in the human saliva and shown to impact the perception of the metabolized aromatic aglycone [18]. A similar observation was brought for another microbial enzyme family, namely, β C-S lyases (β-CSL) [16,26].

β-CSLs catalyze the dissociation of β carbon–sulfur bonds, leading to the release of sulfur aromatic compounds [26,27,28]. Sulfur compounds play a pivotal role as flavor components, often characterized by their remarkably low detection thresholds, and they frequently leave a distinct sensory mark on food products [29]. β-CSLs from plant or microorganisms act on specific aroma precursors found in foods such as onion, garlic, and fruits but also wine and beer to generate sulfur aromatic compounds [27,30,31]. These enzymes are present in plant-based foods and fermentative microorganisms but are also found within the bacteria of the oral cavity, making them significant contributors to flavor at various points along the food production and consumption continuum. For example, specific yeasts harboring enhanced β-CSL activities have been designed for the improved release of sulfur aroma in wine and beer processes [31,32]. Orthologous enzymes found in oral anaerobe bacteria such as *Fusobacterium nucleatum*, were shown to be able to generate sulfur aroma compounds in the mouth during food consumption [26,33,34]. Gaining insights into the biochemistry of β-CSLs appears interesting for both the biotechnological aspects of flavor improvement as well as our understanding of the molecular mechanisms occurring in the oral cavity and impacting flavor perception. This review focuses on the advances made in the field of β-CSLs in relationship with flavor production, encompassing the sulfur compounds produced in plant-based products, in fermented food microorganisms, as well as in the oral cavity.

## 2. β C-S Lyases’ Roles and Structures in Microorganisms

### 2.1. Mechanism and Role in the Sulfur Amino Acid Metabolism

**Figure 1:**
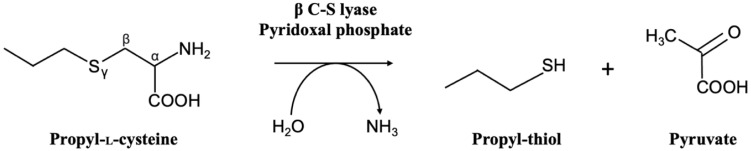
Enzymatic reaction catalyzed by β-CSLs with the example substrate propyl-L-cysteine.

β-CSL, also known as cystathionine β-lyases (EC 4.4.1.8) belong to the larger family of carbon–sulfur lyases, which encompass numerous enzymes capable of dissociating carbon–sulfur bonds such as cystathionine γ-lyase, methionine γ-lyase, or cysteine desulfurase [35,36]. β-CSLs catalyze the dissociation of bonds between the atoms C_β_ and S_γ_. In mammals, including humans, β-CSLs have roles in the metabolism of naturally occurring sulfur and selenium-containing compounds, xenobiotics, and anticancer agents [35]. In bacteria, they are encoded by *metC*, *malY*, or *patB* genes. The *metC* gene encodes cystathionine β-lyase involved in the methionine biosynthetic pathway [37]. The *malY* gene encodes a bifunctional enzyme showing both cystathionine β-lyase activity and a negative activity on regulon maltose [38]. The *patB* gene encodes an enzyme with both cystathionine β-lyase and cysteine desulfhydrase activities [39]. The corresponding enzymes of these genes use the pyridoxal 5′-phosphate (PLP) as cofactor to catalyze β-elimination reactions with L-cysteine or L-cysteine derivatives (such as L-cystathionine, S-methyl-L-cysteine, or longer alkylated cysteines) to form the corresponding thiol compounds with pyruvate and ammonia as end-products (Figure 1). The cofactor PLP, which is the active form of its precursor vitamin B6, is covalently attached to the active site lysine residue conserved in this enzyme family [39].

PLP participates in the enzymatic mechanism by switching from a form where it is bound to a conserved lysine residue (named external aldimine) to a form where PLP is then attached to the amino acid substrate (named internal aldimine) [28]. At the end of the reaction, the β-CSL is again in its external aldimine form and can start a new cycle. β-CSLs were shown to be involved in the metabolism of sulfur amino acid and notably in the transsulfuration pathway. This pathway enables the interconversion between cysteine and methionine in both ways. In humans, the reverse transsulfuration pathway allows the conversion of methionine to cysteine. In bacteria such as *E. coli* or *B. subtilis*, β-CSLs play a role in the forward transsulfuration pathway enabling the conversion of cysteine to methionine. Bacterial β-CSLs react with the intermediate compound cystathionine to form homocysteine, which is a precursor of methionine (further formed by methionine synthase). In addition, several CSL were shown to have a wider spectrum of activity and can also catalyze the β-elimination of other amino acids such as L-cysteine, L-cystine, or longer alkylated cysteines such as S-methyl-, S-ethyl-, or S-propyl-L-cysteine (Figure 1) [37,40,41].

### 2.2. Production of Volatile Sulfur Compounds by Oral Bacteria β-CSL

In addition to their role in sulfur amino acid metabolism, some oral bacterial β-CSLs release volatile sulfur compounds (VSCs). Hydrogen sulfide (H_2_S) is a notable VSC contributing to the malodor associated with periodontal diseases. Many oral bacteria enzymes were described to produce H_2_S, some of them being β-CSLs with a desulfhydrase activity using cysteine as substrate. *Treponema denticola*, an oral pathogen, is involved in periodontitis, and the β-CSL also named cystalysin is considered the main virulence factor of this disease. H_2_S is responsible for the lysis of the cell membrane of erythrocytes (haemolysis), participating in the virulence of the infection [42]. The biochemical characterization of cystalysin from *Treponema denticola* [43,44,45] showed that this enzyme uses the classical PLP cofactor and cysteine to produce H_2_S. The H_2_S release in the mouth is also responsible for halitosis (bad breath) [46]. Other oral bacteria can produce VSCs using β-CSLs. For example, a C-S lyase from *Fusobacterium varium* was purified and its characterization was performed with various alkyl-cysteine conjugates, all being metabolized [47]. In *Fusobacterium nucleatum*, one of the main producers of sulfur volatiles in the mouth, several sulfur-producing enzymes have been described [33]. A recent study showed that the gene *fn0625* encodes a β-CSL enzyme able to produce sulfur volatile compounds from substrates including L-cysteine, L-cystine, S-ethyl-L-cysteine, S-methyl-L-cysteine, L-cystathionine, and DL-lanthionine, with a preference for L-cystine and L-cystathionine [34]. Additionally, the subspecies *F. nucleatum animalis* presents a β-CSL called FnaPatB1 with high activity towards cysteine conjugates harboring hydrophobic substituents, with this high activity being likely due to an elongated active site loop [26].

### 2.3. Generation of Odorant Thiol in the Human Sweat by Bacterial β-CSL

Some bacterial β-CSLs are involved in the production of odorant thiols responsible for sweat odors. For example, *Corynebacteria* present in the axillary region of the human skin express β-CSLs [48,49]. A recent study reported the involvement of a particular class of β-CSL exhibiting a specific activity for thio-alcohol precursors in *Staphylococcus hominis* [28]. These enzymes are a particular class of β-CSL (C-T lyase) whose genes have been horizontally transferred approximately 60 million years ago and became part of an exclusive monophyletic cluster of odor-forming staphylococci. Notably, introducing this enzyme alone into staphylococci that do not produce odor results in the acquisition of odor production, indicating that the C-T lyase is both essential and adequate for thioalcohol formation. By examining the structure of the C-T lyase alongside related enzymes, it becomes apparent that the adaptation to thioalcohol precursors has developed by altering the binding site, creating a restricted hydrophobic pocket with a preference for branched aliphatic thioalcohol ligands. The ancient acquisition of this enzyme and its subsequent evolution in specificity for thioalcohol precursors suggest that the production of body odor in humans is a longstanding biological process [28]. Similar mechanisms may exist in other bacteria and notably the ones of the oral cavity, as some β-CSLs having enhanced activity with branched cysteine derivatives were also identified in anaerobes such as *Fusobacterium nucleatum* [26].

### 2.4. Other Biological Functions of Bacterial β-CSL

In *Escherichia coli*, the *malY* gene encodes a β-CSL able to catalyze classical β-elimination with cysteinyl substrates but also shows the property to act as a repressor of the maltose regulon [50]. The maltose regulon in *E. coli* consists of several genes involved in the metabolism and uptake of maltose and maltodextrins. MalT is the gene activator of the maltose regulon and expression of *malY* represses all MalT-dependent genes. MalY has a cystathionase activity that is not required for its repressing activity on the maltose system. Analysis of MalY crystal structure revealed that amino acids important for repression activity are located far away from the active site and suggests that direct protein–protein interactions are involved between MalY and MalT [37]. Recently, the cryoelectron microscopy structure of the complex formed between MalY and MalT showed that MalY inactivates MalT by blocking its oligomerization and by strengthening ADP-mediated MalT autoinhibition [51]. These results suggest that β-CSLs could have alternative unexplored biological roles involving protein–protein interactions, in addition to their usual C-S lyase activity. They could also have additional catalytic activities. In *Lactobacillus sakei*, Kato and Oikawa identified and characterized Ls-MalY as a bifunctional enzyme harboring both β C-S lyase activity and racemase activity on various amino acids [52]. The PLP cofactor was found to be necessary for both activities occurring in the same active site, suggesting catalytic promiscuity.

### 2.5. Tridimensional Structure of Microbial β-CSLs

**Figure 2:**
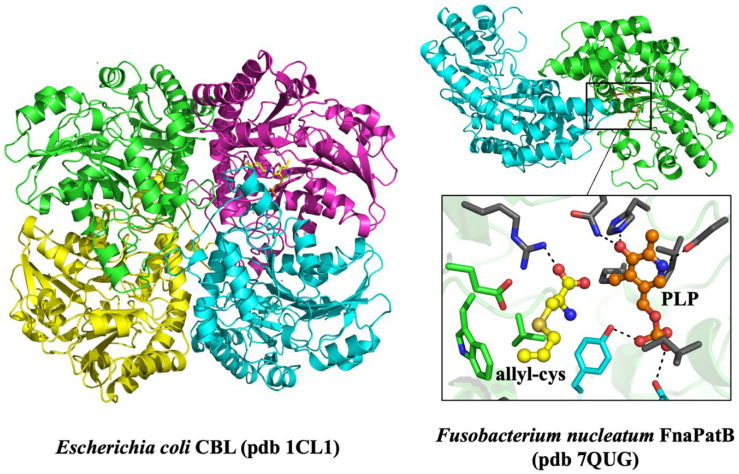
3D structures of β-CSLs from bacteria: heterotetrameric CBL from *E. coli* and heterodimeric FnaPatB1 from *F. nucleatum*, with an enhanced view of its active site bound to S-allyl-L-cysteine and PLP.

β-CSLs whose three-dimensional structures have been determined to date (all by X-ray crystallography) adopt the type-I fold among the PLP-dependent aminotransferases family. This fold consists in homodimeric or homotetrameric assemblies, although β-CSLs determined to date revealed all a homodimeric fold except for the one identified in *E. coli* (Table 1, Figure 2). The monomer of β-CSLs is characterized by a three-domain fold, exemplified by the structure of *E. coli* CBL (EcCBL, pdb 1CL1) [37]. The N-terminal domain, from residues 1 to 60, is made of three α-helices and one β-strand. This domain is part of the active site of the neighboring subunit, and in the case of tetrameric CBL such as *E. coli* CBL can favor tetramer interaction. The second domain, from residues 61 to 256, has an α/β-structure with a central β-sheet and the conserved Lys210 covalently bound to PLP. The C-terminal domain (from residues 257 to 395) is made of four helices surrounding an antiparallel four-stranded β-sheet. β-CSL enzymes with dimeric structure share this fold globally but with some differences notably in the dimerization interface, which despite some structural homology make them fall in a different subfamily within the type-I PLP enzymes [38].

For both oligomeric structures of β-CSL, the active site is made of residues forming a buried pocket where both the PLP cofactor and the amino acid substrate can bind (Figure 2). The PLP cofactor is stabilized deep inside the structure by several hydrogen bonds with conserved amino acids, in addition to a covalent link with the Lys residue involved in the catalytic mechanism, conserved among all PLP-dependent enzymes. The site of the amino acid substrate is located very near from the PLP site but is more accessible to the solvent. It is located in the vicinity of the variable region loop located 20 residues starting from the N-terminal extremity. In the case of FnaPatB1 and ShPatB, this loop is elongated and was suggested to promote favorable hydrophobic contact to stabilize cysteine conjugates with hydrophobic substituents [23,25].

## 3. Substrates of Microbial β C-S Lyases in Food: Cysteine S-Conjugates As Sulfurous Aroma Precursors

Volatile sulfur compounds have low detection thresholds and play a substantial role in creating the distinctive fragrance of animal products such as cheese and meat [54], plant foods such as vegetables belonging notably to *Brassica* and *Allium* categories, as well as mushrooms [29] and fruits [55]. This sets them apart as a particularly distinct category of plant metabolites, intriguing not only from a flavor chemistry perspective but also in terms of analytical considerations, given their wide variety, low odor thresholds, susceptibility to instability, and reactivity. Sulfur compounds, when considered as metabolic byproducts, exhibit diverse chemical configurations. These compounds are found in food and can be generated or modified through enzymatic and chemical reactions. A category of sulfur aroma precursors stands for its interest in flavor generation: cysteine S-conjugates, which are substrates of β-CSL.

Cysteine S-conjugates have been historically studied for their occurrence in *Allium* species (including onion, garlic, and leeks) and are responsible for the characteristic odor of garlic and onions [56]. Cysteine conjugates represent a significant category of precursors for sulfur-based aromas [16]. They contain a cysteine group connected to an organic group through a carbon–sulfur bond. These compounds lack noticeable odors because they have low volatility (considering their soluble nature as amino acids). However, when β-CSLs metabolize these compounds (either in the plant or by microorganisms), they transform into molecules with a free thiol group and distinct aroma characteristics. These precursors are actually present in various plant-based foods additionally to *Allium* species such as *Brassica* (including cauliflower and broccoli), asparagus, bell peppers, and fruits (including passion fruit, guava, and durian). They are also present in beverages like tea, coffee, wine, sake, and beer due to their presence both in hop and in some malts [16,57,58,59,60,61,62,63,64]. Cysteine conjugates can be formed during cooking through Maillard reactions between cysteine and sugars. As such they are expected in a variety of cooked food including cooked meat and coffee [65,66]. For a recent review on sulfur flavor compounds and cysteine conjugates, the reader is referred to the article from Bonnaffoux and coworkers [67]. Cysteine conjugates exhibit a wide chemical diversity at their S-substituent including aliphatic (saturated or unsaturated), aromatic, or S-oxide groups, and consequently this diversity also occurs on the odorant metabolites formed from these precursors (Table 2). The flavor descriptors of the thiol metabolites range from garlic and onion (e.g., for allyl-thiol, propyl-thiol) to catty, boxtree (e.g., 4-mercapto-4-methylpentan-2-one), meaty (2-methyl-3-furanthiol), or coffee (e.g., 2-furfurylthiol) aromas.

In plants, cysteine conjugates are products of the glutathione pathway. As an example, the synthesis of the flavor precursor S-3-(hexan-1-ol)-L-cysteine (cys-3MH) has been described previously [68]. The synthesis of the glutathione conjugate of 3MH occurs after the conjugation of glutathione to trans-2-hexanal with the involvement of glutathione transferase, and subsequent action of γ-glutamyl transferase and carboxypeptidase, to yield the corresponding cysteine conjugate. This pathway was shown to be modulated by environmental factors like ultraviolet irradiation and water deficit, suggesting that flavor precursor concentrations could be modulated by the geographical region, the specific climate, and the culture condition in which plants were grown. In this context, it was observed that hop cultivars from different growing regions showed different concentrations of S-4-(4-methylpentan-2-one)-L-cysteine (4MMP), mainly due to the usage of copper sulfate as a fungicide that decreases the aromatic thiol potential of hops [69]. Another factor influencing the concentration of cysteine conjugates in grapes is the development of the noble-rot *Botrytis cinerea* on the berries. *B. cinerea* was shown to boost the production of the 3-sulfanyl-hexanol precursor in a model of *Vitis vinifera* cell culture 1000-fold [70].

Concerning malts, evidence was shown of enzymatic and chemical interconversions between 3-sulfanyl-hexanol conjugates during mashing [62]. In addition to cysteine S-conjugates, other sulfur precursors exist as glutathione, γ-glutamyl-cysteine, and cysteine-glycine states, while their biotransformation by β-CSLs is, to our knowledge, not well documented. Importantly, some precursors have been historically shown to be metabolized directly by plant enzymes. The best example is given by the β-CSL named alliinase and its substrate S-allyl-L-cysteine. They are separated in the plant cell but they yield the corresponding metabolite allyl-thiol when mixed together when fresh garlic or onion are cut [71]. Other edible plants or mushrooms were shown to contain C-S lyases important for flavor generation such as seeds of *Parkia speciosa* Hassk [72] and *Lentinula edodes* [73,74].

**Table 2:** Aroma precursors biotransformed by β C-S lyases from fermentative microorganisms.

Substrate	Metabolite	Organism/Enzyme	Food Product	References
S-4-(4-methylpentan-2-one)-L-cysteine	4-mercapto-4-methylpentan-2-one	β-CSL from *S. cerevisiae*, *E. limosum*	Sauvignon, Semillon, Chardonnay, Riesling wine	[59,75]
S-4-(4-methylpentan-2-ol)-L-cysteine	4-mercapto-4-methylpentan-2-ol			
S-3-(hexan-1-ol)-L-cysteine	3-mercaptohexan-1-ol			
S-2-(3-methylbutanol)-L-cysteine	2-mercapto-3-methylbutanol	β-CSL from *S. cerevisiae*, *P. kluyveri*	Lager beer	[76,77]
S-1-(3-pentanone)-L-cysteine	1-mercapto-3-pentanone			
S-3-(3-methylbutanol)-L-cysteine	3-mercapto-3-methylbutanol			
S-3-(3-methylbutanol)-L-cysteine	3-mercapto-3-methylbutanol	β-CSL from *E. limosum*	Passion juice	[78]
S-3-(hexan-1-ol)-L-cysteine	3-mercaptohexan-1-ol			
S-furfuryl-L-cysteine	furfurylthiol	STR3 and CYS3 from *S. cerevisiae*	Baijiu, coffee	[65,66,79]
L-cysteine and benzaldehyde	benzenemethanethiol	Yeast β-CSL	Baijiu Daqu	[80]

## 4. Importance of β C-S Lyases in Microorganisms Used for Fermented Food Production

### 4.1. Wine

In the context of wine, specific sulfur compounds are a result of the metabolism of cysteine conjugates by microorganisms having β-CSLs utilized in the fermentation process. Tominaga et al. identified some of these compounds in Sauvignon white wine in 1998, including 4-mercapto-4-methylpentan-2-one, 4-mercapto-4-methylpentan-2-ol, and 3-mercaptohexan-1-ol [59]. These compounds are associated with the typical passion/boxtree aromatic notes of Sauvignon wines and originate from the hydrolysis of nonvolatile cysteinyl precursors present in grape must after being metabolized by yeast. The generation of thiols is facilitated by β-CSLs, which catalyze the dissociation of C-S bonds. Precursors have been identified in various grape varieties such as Semillon, Chardonnay, and Riesling [75]. The corresponding flavor compounds are noteworthy as they contribute to enhancing aroma parameters during wine consumption, including complexity, intensity, and persistence [81]. Some compounds, like S-3-(hexan-1-ol)-L-cysteine, are produced in grapevines through the catabolism of glutathione precursors like S-3-(hexan-1-ol)-glutathione [60], synthesized in grapevines by detoxification systems such as glutathione transferases [22].

### 4.2. Beer

Polyfunctional thiols derived from cysteine-conjugated precursors, such as 3-methyl-2-buten-1-thiol, 2-mercapto-3-methylbutanol, and 3-mercapto-3-methylbutanol, have been identified in Lager beer [76]. These precursors are naturally present in various hop varieties [61] and are likely synthesized by enzymes from the glutathione transferase family [82], similar to the process in grapevines. It has been demonstrated that introducing a commercial aminotransferase with C-S lyase activity (such as tryptophanase from *E. coli*) to a hop solution can induce the formation of free thiols [61]. In beer, their formation occurs during alcoholic fermentation, driven by the enzymatic action of C-S lyases from *S. cerevisiae* or other yeasts. In this context, a *Pichia kluyveri* strain has been patented for improving thiol levels during beer fermentation [83]. Additionally, Belda et al. reported the identification of several *Saccharomyces* and non-*Saccharomyces* yeast strains, such as *Torulaspora delbrueckii*, *Meyerozyma guilliermondi*, and *Kluyveromyces marxianus*, capable of enhanced thiol release through increased lyase activity. These properties are conferred by the presence of a full-length *IRC7* gene, encoding an active β-CSL, contrary to most yeast species that harbor only a truncated version of the gene [32]. While the release of polyfunctional thiols is desirable for improving beer flavor, it has been shown that the presence of polyphenols and an acidic environment can hamper the activity of C-S lyase during fermentation [84]. Current research aims to address these challenges and determine optimized processes for increasing thiol compounds in beer [62]. Alternative methods include adding fermented beer powder made from Sauvignon Blanc grape skins rich in precursors (e.g., Phantasm™ Powder).

### 4.3. Baijiu

Baijiu is a traditional alcoholic beverage produced from the solid-state fermentation of cereals. It is associated with more than 2000 aroma compounds, with the most important being the sulfur aroma compounds such as 2-furfuryl thiol and benzenemethanethiol [85]. These two sulfur aroma compounds are produced from their corresponding cysteinylated precursors, which are metabolized by yeast β-CSL. 2-Furfuryl thiol is responsible for the typical sesame flavor of baijiu. This aroma compound is produced in two steps: (i) furfural and cysteine react together to form the conjugate, and (ii) the conjugate is transformed in 2-furfuryl thiol under the action of yeast STR3 and IRC7 enzymes [79]. A similar mechanism was observed in Baijiu Daqu for benzenemethanethiol, which is produced by yeast from cysteine and benzaldehyde as precursors, followed by the action of yeast β-CSLs [80].

### 4.4. Cheese

Volatile sulfur compounds are important for the flavor of cheese, with methionine as the main aroma precursor. In this regard, two compounds stand for their importance to cheese flavor generation, i.e., methanethiol and dimethyldisulfide. This reaction driving the cheese flavor is mainly catalyzed by methionine γ-lyases (MGLs) in an array of lactic acid bacteria [54] but can also be catalyzed by β-CSLs [86]. A β-CSL was isolated from the strain *Lactococcus lactis* subsp. *cremoris* B78 used for the production of Gouda cheese [87]. This enzyme is able to produce methanethiol from methionine due to a γ-elimination reaction. In *Lactobacillus helveticus* CNRZ 32, overexpression of a β-CSL resulted in higher VSC production from methionine as substrate in a model system, suggesting a similar mechanism [88].

### 4.5. Genes and Corresponding β-CSLs of Interest in Thiol Release Improvement

As stated above, several yeasts and bacteria are used for their CSL activity to improve the flavor of foods. The main yeast genes responsible for β-CSL activity are *IRC7* [89] and *STR3* [77]. These genes encode cystathionine β-lyases and pyridoxal-5′-phosphate-dependent enzymes catalyzing the dissociation of the C-S bond in various substrates, including L-cystathionine, L-cysteine, L-cystine, and precursors of thiol aroma compounds such as 4-sulfanyl-4-methylpentan-2-one and 3-sulfanylhexanol [69]. Enhancing the release of varietal thiols to improve wine flavor can be achieved by engineering strains for the overexpression of these genes [77,89]. Concerning the enzyme encoded by the *IRC7* gene, studies have shown that two forms exist, the full-length IRC7p retaining full activity and a shorter form that is inactive. Few *Saccharomyces* strains have the fully active form, while most of the lab strains including *Saccharomyces cerevisiae* S288C have an inactive form of the *IRC7* gene that is deprived of the last 38 base pairs [31]. Consequently, the yeast strains that harbor only the truncated form have a reduced potential for thiol release, confirming IRC7p as an interesting target for thiol release [32]. STR3p is a β-CSL with a peroxisomal targeting peptide, and an engineered strain having an extra copy of the *STR3* gene under the PGK1 promoter proved to efficiently improve the thiol release [77]. Additionally, two other genes in yeast were found to improve the release of varietal thiols when overexpressed, i.e., *GLO1* and *CYS3* [89]. However, these two genes do not encode true β-CSLs. *GLO1* encodes glyoxalase and *CYS3* encodes a cystathionine γ-lyase [90]. Overexpression of these genes could indirectly improve the thiol release by modifying the yeast metabolism.

The presence of β-CSLs in lactic bacteria, e.g., *Lactobacillus* species [90], suggests potential applications for improving the flavor of specific red wines undergoing malolactic fermentation, as well as other food products. In the subspecies *bulgaricus* of *Lactobacillus*, a β-CSL (LDB) was identified, with broad substrate specificity toward sulfur-containing amino acids [27]. Its characterization revealed that LDB was able to cleave cysteinylated substrate precursors into the corresponding flavor-contributing thiols, with a catalytic efficiency higher than L-cystathionine. *Lactobacillus plantarum* was also suggested to be a useful tool to enhance thiol release in fermented beverages, although the corresponding β-CSL was not studied yet. Takase and coworkers demonstrated that this strain can improve the 3-sulfanyl hexanol release from its precursors, including its glutathione conjugate form, in fermented grape juices [91]. Previously, the introduction of tryptophanase from *E. coli* to a hop solution induced the formation of free thiols [61]. More recently, Clérat and coworkers discussed in more detail the possibility of using exogenous β-CSLs to improve the release of aromas in wine or beer [92]. Using *Lactobacillus delbrueckii* LDB as an example they show a method to follow the activity of the enzyme in presence of various competing cysteine S-conjugate analogs. Due to constraints in terms of pH, LDB has the potential for aroma increase in beer processes but it is not suitable for winemaking, suggesting the need for further research to identify new targets suitable for the wine processes. Additionally, international regulations regarding the use of exogenous β-CSLs for food production are currently lacking and more studies are warranted. More generally, and according to the Food and Agriculture Organization (FAO), the use of enzymes in food for flavor enhancement involves ensuring non-toxicity and GRAS status, assessing allergenicity, and sourcing from acceptable microbial, plant, or animal origins. Purity and consistent enzymatic activity are crucial, alongside regulatory approval and proper labeling. Enzymes must be specific to their substrates, stable under processing conditions, and effective in enhancing the desired flavor profile without off-flavors. In particular, selected β-CSLs should be specific toward small compound precursors and should not react with the cysteine residues of food proteins, in order to preserve them from alteration or aggregation [93].

## 5. Involvement of Oral Bacteria β-CSLs in the In-Mouth VSC Release and Perception

### 5.1. Studies on Pure Compounds

The role of the oral microbiota and its relationships with chemoperception is an increasingly studied topic of research [12]. The oral cavity is colonized by over 700 microbial species, including bacteria, fungi, and viruses [94]. A pioneer work in 2008 led by Starkenmann and coworkers showed that cysteine S-conjugates, the same family of precursors metabolized by microbial enzymes during fermentation, are transformed into their corresponding thiol aromatic compounds under the action of human saliva (Table 3) [16]. This process occurs within a few seconds after the cysteine conjugate precursors are put into the mouth, suggesting that enzymatic mechanisms are compatible with the time of flavor perception. In the same study, Starkenmann et al. showed that the enzymatic reactions of the transformation of cysteine conjugates into thiols are mediated by the oral microbiota. The compounds studied included S-(R/S)-3-(1-hexanol)-L-cysteine, S-(1-propyl)-L-cysteine, and S-((R/S)-2-heptyl)-L-cysteine. Starkenmann and coworkers showed that anaerobic bacteria in saliva, notably *Fusobacterium nucleatum*, are mainly involved in the enzymatic degradation of these precursors, while the appearance of the corresponding thiols increases through time [16]. In humans, odorant thiols are perceived at very low detection thresholds with the involvement of the olfactory receptor OR2T11 [95]. Importantly, *Fusobacterium* spp. possesses an extensive enzymatic repertoire facilitating the metabolism of various sulfur compounds, including cysteine conjugates [33]. More recently, it was shown that human saliva is able to release allyl-thiol, allyl-sulfide, and allyl-disulfide from the precursor compound S-allyl-L-cysteine. Centrifugation of saliva leading to the removal of the saliva pellet containing microbial cells resulted in no metabolization of precursors. Interestingly, the biotransformation was shown to be upregulated by adding the cofactor PLP, while adding a transaminase inhibitor, L-cycloserine, resulted in a drastic decrease in metabolization. Both observations suggest the involvement of β-CSLs from oral bacteria. A subsequent comparative genomic approach identified FnaPatB1 as a putative β-CSL in *F. nucleatum*, which after recombinant production showed high activity with various cysteine S-conjugates precursors and produced the same metabolites as human saliva (Table 3) [26].

### 5.2. Studies on Food Products

In addition to studies on pure compounds, studies on real foods showed the importance of oral β-CSL activity for perception. As previously mentioned, cysteine conjugates exist in various foods [55,67], and all could be potential substrates for oral microbiota β-CSLs with an impact on flavor perception. Two studies led by Frank and coworkers showed the ability of human saliva to metabolize S-Methyl-Cysteine-Sulfoxide (SMCSO) present in raw cabbage and broccoli into its corresponding metabolites methanethiol and dimethyl-disulfide using proton transfer reaction mass spectrometry (PTR-MS). Differences in the rates of C-S lyase activity present in human saliva and associated sulfur volatile production have been shown, with more than 10-fold differences between individuals [96]. A garlic odor was positively correlated with the release of sulfur aroma compounds. Furthermore, the accumulation of sulfur volatiles originating from SMCSO during more than twenty consecutive mouthfuls was observed, which suggests that these reactions have the potential to influence sensory perception during the act of eating.

In a second ex vivo study, volatile production from Brassica vegetables and associations with liking in an adult/child cohort were explored [58]. Production of volatiles was measured within minutes after raw cauliflower was incubated with fresh human saliva, in accordance with the in vivo approach employed during the first study. Substantial variations were observed in the rate at which sulfur volatiles were generated among different individuals, with no notable distinctions noted between various age groups. Notably, strong positive associations were identified in volatile production between adults and children paired together, indicating shared characteristics in saliva composition and oral microbiome activity. Noteworthy negative correlations were identified between the quantity of sulfur volatile production within the mouth and children’s preference for raw cauliflower. Saliva was suggested to be able to trap thiols, probably through salivary proteins [16]. Additionally, several salivary parameters including the salivary antioxidant capacity could be responsible for the modulation of thiol release as it was shown for other reactions in saliva, with a final impact on flavor perception [10].

## 6. Conclusions

β-CSLs are enzymes with important functions in flavor at multiple points along the food continuum. On the one hand, gaining insights into β-CSLs from fermentative microorganisms appears necessary for improving the thiol release in an array of fermented food. On the other hand, the characterization of this enzyme family in oral microorganisms is also very interesting for increasing our understanding of the molecular mechanisms occurring in the mouth. As thiols are associated with the bad taste of vegetables, strategies to inhibit the C-S lyase activity to limit the oral production of thiol could be valuable for increasing the consumption of green vegetables, especially in children. Molecules from food, especially plant molecules, are potent inhibitors of C-S lyases, and the molecular tools from biochemistry and structural biology can help to identify such inhibitors [26]. In the same way, gaining insights into the inhibition of these enzymes could leverage a problem in the usage of these enzymes as flavor enhancers, because they are inhibited by plant molecules such as polyphenols [84]. While the functional and structural characterization of β-CSLs both from oral bacteria or fermentative microorganisms is still important for gaining knowledge on fundamental biochemical processes, the characterization of their activity in a physiologically/biotechnologically relevant context is even more challenging. However, this challenge is necessary to have a more exhaustive view of the enzymatic processes in the context of (i) an oral environment that modulates the enzymatic activities (salivary proteins, pH, redox status) and (ii) a fermentative process including several steps with different biochemical conditions, whether the β-CSL is present in the microorganism used for fermentation or added exogenously.

## Figures and Tables

**Table 1:** Solved X-ray crystallographic structures of microbial β-CSLs available in the PDB. NP means no publication associated with the PDB deposition.

Name	Organism	PDB Code	Resolution (Å)	Oligomerization	References
BaCBL	*Bacillus anthracis*	3T32	2.00	Homodimer	NP
BsPatB	*Bacillus subtilis*	6QP3	2.30	Homodimer	[39]
CdCBL	*Clostridium difficile*	4DGT	1.55	Homodimer	NP
CdCBL	*Corynebacterium diphtheriae*	3FDB	1.99	Homodimer	NP
EcCBL	*Escherichia coli*	1CL1	1.83	Homotetramer	[37,53]
EcMalY		1D2F	2.50	Homodimer	[38]
FnaPatB1	*Fusobacterium nucleatum subsp. animalis*	7QUG	2.60	Homodimer	[26]
KpCBL	*Klebsiella pneumonia*	8DUY	1.90	Homodimer	NP
LDB CSL	*Lactobacillus delbrueckii subs. bulgaricus*	3DZZ	1.61	Homodimer	[27]
ShPatB	*Staphylococcus hominis*	6QP2	1.60	Homodimer	[28]
Cystalysin	*Treponema denticola*	1C7N	1.90	Homodimer	[42]

**Table 3:** Aroma precursors biotransformed by β C-S lyases from saliva/oral microbiota.

Substrate	Metabolite	Organism/Enzyme	Foods/Compounds	References
S-3-(hexan-1-ol)-L-cysteine	3-mercaptohexan-1-ol	Human saliva β-CSL, *F. nucleatum* β-CSL	Pure compounds	[16]
S-(1-propyl)-L-cysteine	1-propanethiol			
S-((R/S)-2-Heptyl)-L-cysteine	2-heptanethiol			
S-methyl-L-cysteine-oxide (Methiin)	methanethiol, dimethyl disulfide, dimethyl trisulfide	Human saliva β-CSL	*Allium* and *Brassica* vegetables	[57,58,96]
S-allyl-L-cysteine	allyl-thiol, diallyl-sulfide, diallyl-disulfide	Human saliva β-CSL, FnaPatB1 from *F. nucleatum*	Pure compounds	[26]

## Data Availability

No new data were created or analyzed in this study.

## References

[1] Heydel J.-M., Coelho A., Thiebaud N., Legendre A., Bon A.-M.L., Faure P., Neiers F., Artur Y., Golebiowski J., Briand L. (2013). Odorant-Binding Proteins and Xenobiotic Metabolizing Enzymes: Implications in Olfactory Perireceptor Events: Odorant-Binding Proteins and Metabolizing Enzymes. Anat. Rec..

[2] Heydel J.M., Hanser H.I., Faure P., Neiers F. (2017). Odorant Metabolizing Enzymes in the Peripheral Olfactory Process. Flavour.

[3] Boichot V., Muradova M., Nivet C., Proskura A., Heydel J.-M., Canivenc-Lavier M.-C., Canon F., Neiers F., Schwartz M. (2022). The Role of Perireceptor Events in FLavor Perception. Front. Food Sci. Technol..

[4] Munoz-Gonzalez C., Brule M., Martin C., Feron G., Canon F. (2021). Molecular Mechanisms of Aroma Persistence: From Noncovalent Interactions between Aroma Compounds and the Oral Mucosa to Metabolization of Aroma Compounds by Saliva and Oral Cells. Food Chem..

[5] Ployon S., Brule M., Andriot I., Morzel M., Canon F. (2020). Understanding Retention and Metabolization of Aroma Compounds Using an in Vitro Model of Oral Mucosa. Food Chem..

[6] Schwartz M., Neiers F., Charles J.P., Heydel J.M., Munoz-Gonzalez C., Feron G., Canon F. (2021). Oral Enzymatic Detoxification System: Insights Obtained from Proteome Analysis to Understand Its Potential Impact on Aroma Metabolization. Compr. Rev. Food Sci. Food Saf..

[7] Robert-Hazotte A., Faure P., Ménétrier F., Folia M., Schwartz M., Le Quéré J.-L., Neiers F., Thomas-Danguin T., Heydel J.-M. (2022). Nasal Odorant Competitive Metabolism Is Involved in the Human Olfactory Process. J. Agric. Food Chem..

[8] Boichot V., Menetrier F., Saliou J.-M., Lirussi F., Canon F., Folia M., Heydel J.-M., Hummel T., Menzel S., Steinke M. (2023). Characterization of Human Oxidoreductases Involved in Aldehyde Odorant Metabolism. Sci. Rep..

[9] Qian W.W., Wei J.N., Sanchez-Lengeling B., Lee B.K., Luo Y., Vlot M., Dechering K., Peng J., Gerkin R.C., Wiltschko A.B. (2023). Metabolic Activity Organizes Olfactory Representations. eLife.

[10] Schwartz M., Neiers F., Feron G., Canon F. (2020). The Relationship Between Salivary Redox, Diet, and Food Flavor Perception. Front. Nutr..

[11] Piombino P., Genovese A., Esposito S., Moio L., Cutolo P.P., Chambery A., Severino V., Moneta E., Smith D.P., Owens S.M. (2014). Saliva from Obese Individuals Suppresses the Release of Aroma Compounds from Wine. PLoS ONE.

[12] Schwartz M., Canon F., Feron G., Neiers F., Gamero A. (2021). Impact of Oral Microbiota on Flavor Perception: From Food Processing to In-Mouth Metabolization. Foods.

[13] López-Dávalos P.C., Requena T., Pozo-Bayón M.Á., Muñoz-González C. (2023). Decreased Retronasal Olfaction and Taste Perception in Obesity Are Related to Saliva Biochemical and Microbiota Composition. Food Res. Int..

[14] Duarte-Coimbra S., Forcina G., Pérez-Pardal L., Beja-Pereira A. (2023). Characterization of Tongue Dorsum Microbiome in Wine Tasters. Food Res. Int..

[15] Xi Y., Yu M., Li X., Zeng X., Li J. (2024). The Coming Future: The Role of the Oral–Microbiota–Brain Axis in Aroma Release and Perception. Comp. Rev. Food Sci. Food Safe.

[16] Starkenmann C., Le Calve B., Niclass Y., Cayeux I., Beccucci S., Troccaz M. (2008). Olfactory Perception of Cysteine-S-Conjugates from Fruits and Vegetables. J. Agric. Food Chem..

[17] Munoz-Gonzalez C., Cueva C., Angeles Pozo-Bayon M., Victoria Moreno-Arribas M. (2015). Ability of Human Oral Microbiota to Produce Wine Odorant Aglycones from Odourless Grape Glycosidic Aroma Precursors. Food Chem..

[18] Parker M., Onetto C., Hixson J., Bilogrevic E., Schueth L., Pisaniello L., Borneman A., Herderich M., de Barros Lopes M., Francis L. (2020). Factors Contributing to Interindividual Variation in Retronasal Odor Perception from Aroma Glycosides: The Role of Odorant Sensory Detection Threshold, Oral Microbiota, and Hydrolysis in Saliva. J. Agric. Food Chem..

[19] Hirst M.B., Richter C.L. (2016). Review of Aroma Formation through Metabolic Pathways of *Saccharomyces cerevisiae* in Beverage Fermentations. Am. J. Enol. Vitic..

[20] Dzialo M.C., Park R., Steensels J., Lievens B., Verstrepen K.J. (2017). Physiology, Ecology and Industrial Applications of Aroma Formation in Yeast. FEMS Microbiol. Rev..

[21] Mao J., Zhou Z., Yang H. (2023). Microbial Succession and Its Effect on the Formation of Umami Peptides during Sufu Fermentation. Front. Microbiol..

[22] Ferreira V., Lopez R. (2019). The Actual and Potential Aroma of Winemaking Grapes. Biomolecules.

[23] Shin K.-C., Nam H.-K., Oh D.-K. (2013). Hydrolysis of Flavanone Glycosides by β-Glucosidase from *Pyrococcus furiosus* and Its Application to the Production of Flavanone Aglycones from Citrus Extracts. J. Agric. Food Chem..

[24] Li S., Tian Y., Jiang P., Lin Y., Liu X., Yang H. (2021). Recent Advances in the Application of Metabolomics for Food Safety Control and Food Quality Analyses. Crit. Rev. Food Sci. Nutr..

[25] Muradova M., Proskura A., Canon F., Aleksandrova I., Schwartz M., Heydel J.-M., Baranenko D., Nadtochii L., Neiers F. (2023). Unlocking Flavor Potential Using Microbial β-Glucosidases in Food Processing. Foods.

[26] Neiers F., Gourrat K., Canon F., Schwartz M. (2022). Metabolism of Cysteine Conjugates and Production of Flavor Sulfur Compounds by a Carbon–Sulfur Lyase from the Oral Anaerobe *Fusobacterium nucleatum*. J. Agric. Food Chem..

[27] Allegrini A., Astegno A., La Verde V., Dominici P. (2017). Characterization of C-S Lyase from *Lactobacillus delbrueckii* Subsp. *Bulgaricus* ATCC BAA-365 and Its Potential Role in Food Flavour Applications. J. Biochem..

[28] Rudden M., Herman R., Rose M., Bawdon D., Cox D.S., Dodson E., Holden M.T.G., Wilkinson A.J., James A.G., Thomas G.H. (2020). The Molecular Basis of Thioalcohol Production in Human Body Odour. Sci. Rep..

[29] Marcinkowska M.A., Jeleń H.H. (2022). Role of Sulfur Compounds in Vegetable and Mushroom Aroma. Molecules.

[30] Chin H.-W., Lindsay R.C. (1994). Mechanisms of Formation of Volatile Sulfur Compounds Following the Action of Cysteine Sulfoxide Lyases. J. Agric. Food Chem..

[31] Roncoroni M., Santiago M., Hooks D.O., Moroney S., Harsch M.J., Lee S.A., Richards K.D., Nicolau L., Gardner R.C. (2011). The Yeast IRC7 Gene Encodes a Beta-Lyase Responsible for Production of the Varietal Thiol 4-Mercapto-4-Methylpentan-2-One in Wine. Food Microbiol..

[32] Belda I., Ruiz J., Navascues E., Marquina D., Santos A. (2016). Improvement of Aromatic Thiol Release through the Selection of Yeasts with Increased Beta-Lyase Activity. Int. J. Food Microbiol..

[33] Basic A., Blomqvist M., Dahlen G., Svensater G. (2017). The Proteins of Fusobacterium Spp. Involved in Hydrogen Sulfide Production from L-Cysteine. BMC Microbiol..

[34] Yoshida Y., Ito S., Kamo M., Kezuka Y., Tamura H., Kunimatsu K., Kato H. (2010). Production of Hydrogen Sulfide by Two Enzymes Associated with Biosynthesis of Homocysteine and Lanthionine in *Fusobacterium nucleatum* Subsp. *Nucleatum* ATCC 25586. Microbiology.

[35] Cooper A.J., Krasnikov B.F., Niatsetskaya Z.V., Pinto J.T., Callery P.S., Villar M.T., Artigues A., Bruschi S.A. (2011). Cysteine S-Conjugate Beta-Lyases: Important Roles in the Metabolism of Naturally Occurring Sulfur and Selenium-Containing Compounds, Xenobiotics and Anticancer Agents. Amino Acids.

[36] Alexander F.W., Sandmeier E., Mehta P.K., Christen P. (1994). Evolutionary Relationships among Pyridoxal-5′-phosphate-dependent Enzymes: Regio-specific α, β and γ Families. Eur. J. Biochem..

[37] Clausen T., Huber R., Laber B., Pohlenz H.-D., Messerschmidt A. (1996). Crystal Structure of the Pyridoxal-5′-Phosphate Dependent Cystathionine β-Lyase fromEscherichia Coliat 1.83 Å. J. Mol. Biol..

[38] Clausen T., Schlegel A., Peist R., Schneider E., Steegborn C., Chang Y.-S., Haase A., Bourenkov G.P., Bartunik H.D., Boos W. (2000). X-Ray Structure of MalY from Escherichia Coli: A Pyridoxal 5′-Phosphate-Dependent Enzyme Acting as a Modulator in Mal Gene Expression. EMBO J..

[39] Auger S., Gomez M.P., Danchin A., Martin-Verstraete I. (2005). The PatB Protein of Bacillus Subtilis Is a C-S-Lyase. Biochimie.

[40] Aitken S.M., Lodha P.H., Morneau D.J.K. (2011). The Enzymes of the Transsulfuration Pathways: Active-Site Characterizations. Biochim. Biophys. Acta BBA Proteins Proteom..

[41] Auger S., Yuen W.H., Danchin A., Martin-Verstraete I. (2002). The metIC Operon Involved in Methionine Biosynthesis in Bacillus Subtilis Is Controlled by Transcription Antitermination. Microbiology.

[42] Krupka H.I., Huber R., Holt S.C., Clausen T. (2000). Crystal Structure of Cystalysin from Treponema Denticola: A Pyridoxal 5′-phosphate-dependent Protein Acting as a Haemolytic Enzyme. EMBO J..

[43] Chu L., Ebersole J.L., Kurzban G.P., Holt S.C. (1997). Cystalysin, a 46-Kilodalton Cysteine Desulfhydrase from Treponema Denticola, with Hemolytic and Hemoxidative Activities. Infect. Immun..

[44] Chu L., Ebersole J.L., Kurzban G.P., Holt S.C. (1999). Cystalysin, a 46-kDa L-Cysteine Desulfhydrase from Treponema Denticola: Biochemical and Biophysical Characterization. Clin. Infect. Dis..

[45] Bertoldi M., Cellini B., Clausen T., Voltattorni C.B. (2002). Spectroscopic and Kinetic Analyses Reveal the Pyridoxal 5′-Phosphate Binding Mode and the Catalytic Features of *Treponema denticola* Cystalysin. Biochemistry.

[46] Krespi Y.P., Shrime M.G., Kacker A. (2006). The Relationship between Oral Malodor and Volatile Sulfur Compound-Producing Bacteria. Otolaryngol. Head. Neck Surg..

[47] Tomisawa H., Suzuki S., Ichihara S., Fukazawa H., Tateishi M. (1984). Purification and Characterization of C-S Lyase from Fusobacterium Varium. A C-S Cleavage Enzyme of Cysteine Conjugates and Some S-Containing Amino Acids. J. Biol. Chem..

[48] Starkenmann C., Niclass Y., Troccaz M., Clark A.J. (2005). Identification of the Precursor of (S)-3-Methyl-3-Sulfanylhexan-1-Ol, the Sulfury Malodour of Human Axilla Sweat. Chem. Biodivers..

[49] Natsch A., Schmid J., Flachsmann F. (2004). Identification of Odoriferous Sulfanylalkanols in Human Axilla Secretions and Their Formation through Cleavage of Cysteine Precursors by a C–S Lyase Isolated from Axilla Bacteria. Chem. Biodivers..

[50] Zdych E., Peist R., Reidl J., Boos W. (1995). MalY of Escherichia Coli Is an Enzyme with the Activity of a Beta C-S Lyase (Cystathionase). J. Bacteriol..

[51] Wu Y., Sun Y., Richet E., Han Z., Chai J. (2023). Structural Basis for Negative Regulation of the *Escherichia coli* Maltose System. Nat. Commun..

[52] Kato S., Oikawa T. (2018). A Novel Bifunctional Amino Acid Racemase With Multiple Substrate Specificity, MalY From *Lactobacillus sakei* LT-13: Genome-Based Identification and Enzymological Characterization. Front. Microbiol..

[53] Clausen T., Huber R., Messerschmidt A., Pohlenz H.-D., Laber B. (1997). Slow-Binding Inhibition of *Escherichia coli* Cystathionine β-Lyase by l-Aminoethoxyvinylglycine: A Kinetic and X-ray Study. Biochemistry.

[54] Landaud S., Helinck S., Bonnarme P. (2008). Formation of Volatile Sulfur Compounds and Metabolism of Methionine and Other Sulfur Compounds in Fermented Food. Appl. Microbiol. Biotechnol..

[55] Cannon R.J., Ho C.T. (2018). Volatile Sulfur Compounds in Tropical Fruits. J. Food Drug Anal..

[56] Li J., Dadmohammadi Y., Abbaspourrad A. (2022). Flavor Components, Precursors, Formation Mechanisms, Production and Characterization Methods: Garlic, Onion, and Chili Pepper Flavors. Crit. Rev. Food Sci. Nutr..

[57] Yamazaki Y., Iwasaki K., Mikami M., Yagihashi A. (2010). Distribution of Eleven Flavor Precursors, S-Alk(En)Yl-L-Cysteine Derivatives, in Seven Allium Vegetables. Food Sci. Technol. Res..

[58] Frank D., Piyasiri U., Archer N., Heffernan J., Poelman A.A.M. (2021). In-Mouth Volatile Production from Brassica Vegetables (Cauliflower) and Associations with Liking in an Adult/Child Cohort. J. Agric. Food Chem..

[59] Tominaga T., des Gachons C.P., Dubourdieu D. (1998). A New Type of Flavor Precursors in Vitis Vinifera L Cv Sauvignon Blanc: S-Cysteine Conjugates. J. Agric. Food Chem..

[60] Peyrot Des Gachons C., Tominaga T., Dubourdieu D. (2002). Sulfur Aroma Precursor Present in S-Glutathione Conjugate Form: Identification of S-3-(Hexan-1-Ol)-Glutathione in Must from *Vitis vinifera* L. Cv. Sauvignon Blanc. J. Agric. Food Chem..

[61] Gros J., Tran T.T.H., Collin S. (2013). Enzymatic Release of Odourant Polyfunctional Thiols from Cysteine Conjugates in Hop. J. Inst. Brew..

[62] Chenot C., Collin S., Suc L., Roland A. (2023). Evidence of Enzymatic and Chemical Interconversions of Barley Malt 3-Sulfanylhexanol Conjugates during Mashing. J. Agric. Food Chem..

[63] Starkenmann C., Luca L., Niclass Y., Praz E., Roguet D. (2006). Comparison of Volatile Constituents of *Persicaria odorata* (Lour.) Soják (*Polygonum odoratum* Lour.) and *Persicaria hydropiper* L. Spach (*Polygonum hydropiper* L.). J. Agric. Food Chem..

[64] Starkenmann C., Niclass Y., Escher S. (2007). Volatile Organic Sulfur-Containing Constituents in *Poncirus trifoliata* (L.) Raf. (Rutaceae). J. Agric. Food Chem..

[65] Cerny C., Guntz-Dubini R. (2013). Formation of Cysteine-S-Conjugates in the Maillard Reaction of Cysteine and Xylose. Food Chem..

[66] Cerny C., Schlichtherle-Cerny H., Gibe R., Yuan Y. (2021). Furfuryl Alcohol Is a Precursor for Furfurylthiol in Coffee. Food Chem..

[67] Bonnaffoux H., Roland A., Schneider R., Cavelier F. (2021). Spotlight on Release Mechanisms of Volatile Thiols in Beverages. Food Chem..

[68] Kobayashi H., Takase H., Suzuki Y., Tanzawa F., Takata R., Fujita K., Kohno M., Mochizuki M., Suzuki S., Konno T. (2011). Environmental Stress Enhances Biosynthesis of Flavor Precursors, S-3-(Hexan-1-Ol)-Glutathione and S-3-(Hexan-1-Ol)-L-Cysteine, in Grapevine through Glutathione S-Transferase Activation. J. Exp. Bot..

[69] Kishimoto T., Kobayashi M., Yako N., Iida A., Wanikawa A. (2008). Comparison of 4-Mercapto-4-Methylpentan-2-One Contents in Hop Cultivars from Different Growing Regions. J. Agric. Food Chem..

[70] Thibon C., Cluzet S., Mérillon J.M., Darriet P., Dubourdieu D. (2011). 3-Sulfanylhexanol Precursor Biogenesis in Grapevine Cells: The Stimulating Effect of *Botrytis cinerea*. J. Agric. Food Chem..

[71] Schwimmer S., Mazelis M. (1963). Characterization of Alliinase of Allium Cepa (Onion). Arch. Biochem. Biophys..

[72] Zhang M., Batra R., Brainta M., Huang D. (2023). Purification and Characterisation of a C-S Lyase in Seeds of Parkia Speciosa Hassk. Food Chem..

[73] Lei X., Gao S., Feng X., Huang Z., Bian Y., Huang W., Liu Y. (2019). Effects of GGT and C-S Lyase on the Generation of Endogenous Formaldehyde in Lentinula Edodes at Different Growth Stages. Molecules.

[74] Wang Y., Bao D.-P., Yang R.-H., Chen H.-Y., Gao Y.-N., Li Y., Mao W.-J., Wu Y.-Y. (2018). Bioinformatics Analyses of C-S Lyases in the Genome of Lentinula Edode. Mycosystema.

[75] Peña-Gallego A., Hernández-Orte P., Cacho J., Ferreira V. (2012). S-Cysteinylated and S-Glutathionylated Thiol Precursors in Grapes. A Review. Food Chem..

[76] Vermeulen C., Lejeune I., Tran T.T., Collin S. (2006). Occurrence of Polyfunctional Thiols in Fresh Lager Beers. J. Agric. Food Chem..

[77] Holt S., Cordente A.G., Williams S.J., Capone D.L., Jitjaroen W., Menz I.R., Curtin C., Anderson P.A. (2011). Engineering *Saccharomyces cerevisiae* to Release 3-Mercaptohexan-1-Ol during Fermentation through Overexpression of an S. Cerevisiae Gene, STR3, for Improvement of Wine Aroma. Appl. Environ. Microbiol..

[78] Tominaga T., Dubourdieu D. (2000). Identification of Cysteinylated Aroma Precursors of Certain Volatile Thiols in Passion Fruit Juice. J. Agric. Food Chem..

[79] Zha M., Sun B., Yin S., Mehmood A., Cheng L., Wang C. (2018). Generation of 2-Furfurylthiol by Carbon–Sulfur Lyase from the *Baijiu* Yeast *Saccharomyces cerevisiae* G20. J. Agric. Food Chem..

[80] Zhang G., Xiao P., Xu Y., Li H., Li H., Sun J., Sun B. (2023). Isolation and Characterization of Yeast with Benzenemethanethiol Synthesis Ability Isolated from Baijiu Daqu. Foods.

[81] Parker M., Capone D.L., Francis I.L., Herderich M.J. (2018). Aroma Precursors in Grapes and Wine: Flavor Release during Wine Production and Consumption. J. Agric. Food Chem..

[82] Eriksen R.L., Padgitt-Cobb L.K., Townsend M.S., Henning J.A. (2021). Gene Expression for Secondary Metabolite Biosynthesis in Hop (*Humulus lupulus* L.) Leaf Lupulin Glands Exposed to Heat and Low-Water Stress. Sci. Rep..

[83] Holt S., Miks M.H., de Carvalho B.T., Foulquie-Moreno M.R., Thevelein J.M. (2019). The Molecular Biology of Fruity and Floral Aromas in Beer and Other Alcoholic Beverages. FEMS Microbiol. Rev..

[84] Chenot C., Willemart G., Gros J., Collin S. (2023). Ability of Exogenous or Wort Endogenous Enzymes to Release Free Thiols from Hop Cysteinylated and Glutathionylated *S*-Conjugates. J. Am. Soc. Brew. Chem..

[85] Song X., Zhu L., Wang X., Zheng F., Zhao M., Liu Y., Li H., Zhang F., Zhang Y., Chen F. (2019). Characterization of Key Aroma-Active Sulfur-Containing Compounds in Chinese Laobaigan Baijiu by Gas Chromatography-Olfactometry and Comprehensive Two-Dimensional Gas Chromatography Coupled with Sulfur Chemiluminescence Detection. Food Chem..

[86] Hanniffy S.B., Peláez C., Martínez-Bartolomé M.A., Requena T., Martínez-Cuesta M.C. (2009). Key Enzymes Involved in Methionine Catabolism by Cheese Lactic Acid Bacteria. Int. J. Food Microbiol..

[87] Alting A.C., Engels W., Van Schalkwijk S., Exterkate F.A. (1995). Purification and Characterization of Cystathionine (Beta)-Lyase from *Lactococcus lactis* Subsp. Cremoris B78 and Its Possible Role in Flavor Development in Cheese. Appl. Environ. Microbiol..

[88] Lee W.-J., Banavara D.S., Hughes J.E., Christiansen J.K., Steele J.L., Broadbent J.R., Rankin S.A. (2007). Role of Cystathionine β-Lyase in Catabolism of Amino Acids to Sulfur Volatiles by Genetic Variants of *Lactobacillus helveticus* CNRZ 32. Appl. Environ. Microbiol..

[89] Howell K.S., Klein M., Swiegers J.H., Hayasaka Y., Elsey G.M., Fleet G.H., Høj P.B., Pretorius I.S., De Barros Lopes M.A. (2005). Genetic Determinants of Volatile-Thiol Release by *Saccharomyces cerevisiae* during Wine Fermentation. Appl. Environ. Microbiol..

[90] Yamagata S., D’Andrea R.J., Fujisaki S., Isaji M., Nakamura K. (1993). Cloning and Bacterial Expression of the CYS3 Gene Encoding Cystathionine Gamma-Lyase of *Saccharomyces cerevisiae* and the Physicochemical and Enzymatic Properties of the Protein. J. Bacteriol..

[91] Takase H., Sasaki K., Kiyomichi D., Kobayashi H., Matsuo H., Takata R. (2018). Impact of Lactobacillus Plantarum on Thiol Precursor Biotransformation Leading to Production of 3-Sulfanylhexan-1-Ol. Food Chem..

[92] Clérat L., Rémond E., Schneider R., Cavelier F., Vivès E. (2024). Exogenous C–S Lyase Enzyme, a Potential Tool To Release Aromas in Wine or Beer?. J. Agric. Food Chem..

[93] Zhu Z., Pius Bassey A., Cao Y., Ma Y., Huang M., Yang H. (2022). Food Protein Aggregation and Its Application. Food Res. Int..

[94] Lamont R.J., Koo H., Hajishengallis G. (2018). The Oral Microbiota: Dynamic Communities and Host Interactions. Nat. Rev. Microbiol..

[95] Li S., Ahmed L., Zhang R., Pan Y., Matsunami H., Burger J.L., Block E., Batista V.S., Zhuang H. (2016). Smelling Sulfur: Copper and Silver Regulate the Response of Human Odorant Receptor OR2T11 to Low-Molecular-Weight Thiols. J. Am. Chem. Soc..

[96] Frank D., Piyasiri U., Archer N., Jenifer J., Appelqvist I. (2018). Influence of Saliva on Individual In-Mouth Aroma Release from Raw Cabbage (*Brassica oleracea* Var. Capitata f. *Rubra* L.) and Links to Perception. Heliyon.

